# Alkaloidal Therapeutics

**Published:** 1889-02

**Authors:** Cassius D. Wescott


### ALKALOIDAL THERAPEUTICS.

BY CASSIUS D. WESCOTT, M. D.

Read before the Chicago Pathological Society September 10, 1888.

GENTLEMEN:—For about a year I have been prescribing the alkaloids, and other active principles of the materia medica, almost to the exclusion of preparations of the entire drug. My results have been more uniformly prompt and certain than by the old method, and in some cases quite remarkable.

I have used samples of nearly all of the pills, tablets and triturates on the market, but freely confess my preference for the granules of the Metric Granule Company, of this city. They are easily taken, are readily soluble, and in many experience positive and uniform in effect.

I am fully satisfied of the desirability of "small doses frequently repeated," for by this means I have frequently produced a desired effect, by the exhibition of a much smaller total quantity of drug than by the use of the ordinary doses at longer intervals.

Among the many happy experiences which I have had, the following are illustrative:

CASE 1. A man, aged 26, street car conductor by occupation, complained of facial neuralgia, due to exposure. I gave him six granules of aconitine, each containing $\frac{1}{500}$ of a grain, one to be taken every half hour while in pain. He reported the next day that the first granule had completely relieved the pain, which had been persistently severe for forty-eight hours.

CASE 2. A lady, aged 32, nervous temperament, complained of super-orbital neuralgia. I ordered six granules of aconitine, $\frac{1}{500}$ of a grain, one to be taken every half hour until relieved. She was completely relieved after taking the second granule. This lady is subject to neuralgia, and has resorted to the usual anodynes and nervines with varying results.

CASE 3. A lady, aged 30, nervous temperament, extensive erysipelas of the face, which had been ushered in by a chill twenty-four hours previous to my call. Temperature 103, pulse 120. I prescribed aconitine, $\frac{1}{500}$ of a grain every hour; atropine, $\frac{1}{250}$ of a grain every two hours. The color of the eruption began to fade in twenty-four hours, and the temperature fell to 101°. I found that by shortening the intervals between the doses of aconitine I could keep the pulse very nearly normal, and make my patient much more comfortable than is usual in such cases. The eruption had entirely disappeared at the end of a week. Locally I used a simple dressing of carbolized cosmoline, and in addition to the above treatment gave Hunyadi water as a laxative, and tonic doses of quinia sulphate after the third day. This patient had had erysipelas before, the eruption lasting three weeks.

CASE 4. A lady, aged 33, erysipelas of the face. I saw this case within a very few hours after its commencement. The nose was characteristically red and swollen, and there was fever and general malaise. I ordered bismuth oleate locally, and gave aconitine $\frac{1}{500}$ of a grain every hour. The inflammation spread during the first twelve hours until it involved the cheeks, but there was no further extension, and in three days it had entirely disappeared. After six doses of the medicine had been taken the interval was doubled, and the aconitine was discontinued after the second day, as the temperature remained normal.

CASE 5. A lady aged 26, complained of the usual symptoms of gastric catarrh. I ordered hydrastine hydrochlorate, one granule of $\frac{1}{34}$ of a grain before meals, without suggesting any restriction as to diet. In three days the patient reported better appetite, better stools, and less distress after taking food. The improvment continued, and the medicine was stopped after ten days.

CASE 6. A lady aged 42, case of spinal irritation of twenty years' duration, with the usual paroxysms of distressing pain. I was able to control the pain with cicutine (conine) better than with anything else tried, without unpleasant after affect of any kind. I gave four granules at a dose, a total of $\frac{1}{34}$ of a grain every fifteen minutes, until relief came, which was usually in less than an hour.

CASE 7. A lady, aged 28, of sedentary habits, complained of frequently desiring to urinate. Examination of the pelvis revealed retroversion of the uterus, but no tenderness due to inflammation of the bladder or its surroundings. The urine was found normal. I prescribed two granules, of gelsemin, a total of $\frac{1}{67}$ of a grain every two hours, which gave perfect relief after a few doses had been taken.

CASE 8. A lady, suffering extremely from nausea and vomiting twelve hours after taking morphine hypodermically. I gave cocaine, $\frac{1}{67}$ of a grain every fifteen minutes. Relief was complete after taking the fourth granule, and the nausea did not return.

CASE 9. A gentleman, in the first stage of phthisis pulmonalis, taking cod liver oil and the hypophosphites, was greatly annoyed by paroxysms of distressing cough. I prescribed codeine, $\frac{1}{67}$ of a grain to be taken every two hours, with the effect of making the cough easy and facilitating expectoration.

CASE 10. A man complaining of constipation, with clay colored stools. I ordered podophyllin, $\frac{1}{6}$ of a grain at bedtime. The first granule gave him a characteristic movement the following morning, and by repeating the dose for a few evenings he was completely relieved of his trouble.—*West. Med. Rec.*

